# Divergence in male sexual odor signal and genetics across populations of the red mason bee, *Osmia bicornis*, in Europe

**DOI:** 10.1371/journal.pone.0193153

**Published:** 2018-02-22

**Authors:** Taina Conrad, Robert J. Paxton, Günter Assum, Manfred Ayasse

**Affiliations:** 1 Institute of Evolutionary Ecology and Conservation Genomics, University of Ulm, Ulm, Germany; 2 Institute of Biology/General Zoology, Martin-Luther-University Halle-Wittenberg, Halle, Germany; 3 Institute of Human Genetics, University of Ulm, Ulm, Germany; Biocenter, Universität Würzburg, GERMANY

## Abstract

In some insect species, females may base their choice for a suitable mate on male odor. In the red mason bee, *Osmia bicornis*, female choice is based on a male’s odor bouquet as well as its thorax vibrations, and its relatedness to the female, a putative form of optimal outbreeding. Interestingly, *O*. *bicornis* can be found as two distinct color morphs in Europe, which are thought to represent subspecies and between which we hypothesize that female discrimination may be particularly marked. Here we investigated (i) if these two colors morphs do indeed represent distinct, reproductively differentiated populations, (ii) how odor bouquets of male *O*. *bicornis* vary within and between populations, and (iii) whether variation in male odor correlates with genetic distance, which might represent a cue by which females could optimally outbreed. Using GC and GC-MS analysis of male odors and microsatellite analysis of males and females from 9 populations, we show that, in Denmark, an area of subspecies sympatry, the two color morphs at any one site do not differ, either in odor bouquet or in population genetic differentiation. Yet populations across Europe are distinct in their odor profile as well as being genetically differentiated. Odor differences do not, however, mirror genetic differentiation between populations. We hypothesize that populations from Germany, England and Denmark may be under sexual selection through female choice for local odor profiles, which are not related to color morph though which could ultimately lead to population divergence and speciation.

## Introduction

Chemical communication is undoubtedly one of the most ancestral forms of signal transfer amongst animals [1]. It plays an especially important role in insects [2], where it fulfils various functions in diverse interactions such as foraging [3], nestmate recognition [2,4], social behavior [5] and mating [6,7]. A wide variety of insects use sex pheromones in diverse ways [7,1]: to attract males, to identify receptive females, and to elicit territorial and courtship behavior in males [6–8]. Females of the moth *Utetheisa ornatrix* Linnaeus (Lepidoptera: Erebidae) use the quantity of the male courtship pheromone hydroxydanaidal to determine a male's size. This pheromone is the sole trait used by a female to select a mate [9].

Odor bouquets also have a function in kin recognition in a range of animals, including bees [10–12,7,13,14]. The most prominent examples of kin recognition in the context of mating come from the sweat bees *Lasioglossum zephyrum* SMITH (Hymenoptera: Halictidae) and *Lasioglossum malachurum* KIRBY (Hymenoptera: Halictidae), in which males distinguish females according to their degree of relatedness using odor cues and preferentially mate with unrelated females [10,11].

In the red mason bee, *Osmia bicornis* Linnaeus (Hymenoptera: Megachilidae), males are attracted by a sex pheromone emitted by the female that elicits intensive courtship behavior [15,16]. Through scramble-competition, one male establishes itself on the female’s venter and then commences precopulatory courtship. Not much is known about possible mate choice in this situation, but it is feasible that males choose a female according to her odor or adjust their courtship according to their own assessment of a female’s suitability.

During precopulatory courtship, the male sits on the female's venter, embracing her from behind. He then vibrates his thorax, rubs himself against the female and passes his antennae repeatedly over those of the female and his forelegs over the female’s compound eyes [15]. The female may reject the male at any time by bending her abdomen away or by physically shaking him off her back. In a previous study, we were able to show that females choose partners based on a male’s odor, his vibrations, and his relatedness to the female [16,17]. Males with longer duration vibrations were chosen by the female, making vibrations a likely signal of vitality. Surprisingly, females were also able to use the males’ vibrations as a signal for country of origin [17] and former investigations revealed that females markedly preferred mating with males from their own region as opposed to males from another country [17]. Genetic analysis showed that females often chose their mating partner according to an optimal outbreeding criterion [18], avoiding mating with males which were either too closely or too distantly related [16]. Odor might encode the degree of kinship, as is the case in *Lasioglossum* [11].

Female choice can be a driving force of speciation if female preference differs between different forms of those traits [19–22]. It is, therefore, plausible that differences in the odor bouquets of *Osmia* males that correlate with kinship might also function as selected traits that lead to the evolution of reproductive isolating barriers between different populations. A similar process has already been shown in the closely related moths *Heliothis zea* BODDIE (Lepidoptera: Noctuidea) and *H*. *virescens* FABRICIUS (Lepidoptera: Noctuidae), albeit in relation to odors emitted by females to attract males. The main component of the female sex pheromone of these moths is (Z)-11-hexadecenal; female *H*. *zea* also use four additional minor components, while female *H*. *virescens* use these and an additional three compounds. These differences function as isolating barriers, and hybridization is prevented [23].

According to Peters [24], *O*. *bicornis* in Europe occurs as two allopatric subspecies: *O*. *bicornis rufa* and *O*. *bicornis cornigera*. The latter subspecies is found in central Europe, whereas *O*. *bicornis rufa* is found around the northern and western edges of Europe (S1 Fig). However, these two subspecies are sympatric in Denmark (S1 Fig). The classification of *O*. *bicornis* into subspecies by Peters [24] is based on a single morphological trait, the color of the hairs at the tip of the abdomen of males and females (red in *O*. *bicornis rufa* and black in *O*. *bicornis cornigera*); color differences may reflect reproductive isolation and may be mirrored in differences among females in male preference. It is, however, important to analyze genetic data in order to draw conclusions about the evolutionary affiliation and degree of isolation of subspecies, particularly where they are sympatric [25,22]. Such genetic data would also allow a test of the correlation between genetic differentiation and odor signals, which could suggest a role for sexual selection in incipient speciation.

Because chemical communication has been relatively well researched in *O*. *bicornis*, as the species is widespread in Europe and relatively abundant (allowing ready access to populations), and because two distinct color morphs have been described (suggesting population differentiation), the species is a potentially valuable model with which to explore the role of odor in reproductive isolation and speciation (e.g. [26]). *Osmia cornuta* Latreille (Hymenoptera: Megachilidae) is a sibling species to *O*. *bicornis* whose phenology is slightly earlier than, though largely overlaps with, that of *O*. *bicornis* [27]. Its distribution also overlaps with *O*. *bicornis* in central Europe, which makes it an ideal species to be used as an inter-specific reference to our intraspecific (between population) analyses of *O*. *bicornis*.

The aim of this study was first to ascertain if there are differences in odor bouquets between the subspecies of *O*. *bicornis*, as defined by Peters [24]. We predicted that the two subspecies of *O*. *bicornis* might differ significantly where they occur in sympatry (in Denmark), if subspecies represent incipient species, whereas populations might be more similar in allopatry (in Germany and England). A further aim was to correlate genetic differentiation within and between populations of both subspecies of *O*. *bicornis* with their odor differences; our prediction was that population pairwise genetic differentiation would correlate with odor differences if drift were the major force shaping both genetic and odor differences.

## Materials and methods

### Study animals

Both *O*. *bicornis* and the closely related *Osmia cornuta* are widespread solitary bees, common in Europe and easily reared in trap nests (e.g. bamboo canes, [15]). Two subspecies of the former, as defined by Peters [24], were used: *O*. *bicornis rufa* and *O*. *bicornis cornigera*.

We used *O*. *bicornis* from natural populations in Germany (Regensburg: 49°0 N 12°6 E (♂n = 71; ♀n = 30), Constance: 47°39 N 9°10 E (♂n = 95; ♀n = 46), Halle: 52°3 N 8°21 E (♂n = 58; ♀n = 67)), England (Kent: 47°22 N 122°14 W (♂n = 81; ♀n = 18), Hereford: 52°3 N 2°42 W (♂n = 22; ♀n = 22), Tonbridge: 51°11 N 0°16 E (♂n = 57; ♀n = 28)), and Denmark (Mön: 55°0 N 12°20 E (♂n = 17; ♀n = 3), Copenhagen: 55°40 N 12°33 E (♂n = 63; ♀n = 22) and Vejle: 55°42 N 9°32 E (♂n = 38; ♀n = 41)) (S1 Fig). *Osmia bicornis cornigera* in Central and Southern Europe is also commonly found together with its sister species, *O*. *cornuta* [24], which it closely resembles. Therefore, we used *O*. *cornuta* from Ulm, Germany, to serve as an out-group. Bees were collected as pupae or pharate adults in cocoons in 2008 and 2009 and emerged in separate flight cages (ca. 29cm x 29cm x 29cm) the following spring. They were provided *ad libitum* with a 50% sugar solution of APIInvert® (Südzucker AG, Germany; 1g citric acid and 3g potassium sorbate were added per litre API-Invert solution) and kept at room temperature under natural light. After observations and experiments, the bees were frozen in liquid nitrogen and kept in the freezer at -20°C for further analysis.

### Chemical analyses

In order to obtain volatiles of the male antennal surface, as this is the surface most in contact with the female antennae, one of the antennae of a male bee was washed in a vial containing 100μl pentane (99%, Sigma Aldrich Chemie GmbH) at +4°C for 24h. The pentane was evaporated under a stream of nitrogen to a volume of 10μl, and an internal standard of 1μg n-undecane added.

To determine the cuticle odor profile of individual male antennae, all samples were analyzed using a gas chromatograph (HP 5890, Series II, Hewlett Packard, Palo Alto, CA) equipped with a FID (flame ionization detector), a nonpolar DB-5 column (30 m x 0.25 mm i.d. x 50 μm film, J&W) and hydrogen (2 ml/min constant flow) as the carrier gas. One μl of a sample was injected splitless at an initial oven temperature of 50°C. After 1 min, the splitting valve was opened and the temperature increased by 10°C/min until it reached 310°C, where it was kept constant for 50 min. To ensure consistency of the analyses, a GC run with a synthetic alkane standard mixture was regularly performed. Structure elucidation of individual compounds, except for the sterols (see [28]), was performed with an HP 6890 gas chromatograph (Hewlett Packard) connected to a mass selective detector (GCMS; Quadrupol 5972, Agilent, Santa Clara, CA, USA). using the methods described in Conrad [16]. Based on previous work [29,30,16,28,31], structure assignments were carried out by comparison of mass spectra and retention times of natural products with corresponding data from synthetic reference samples, using the NIST database and a database of the Institute of Evolutionary Ecology and Conservation Genomics at the University of Ulm. Double-bond positions in alkenes were determined by investigation of the corresponding dimethyl disulfide adducts [32].

We analyzed on average 22 antennae (one per male; 16–25 per population) from each of nine populations, leading to a total of 197 male *O*. *bicornis* samples. Sample sizes for the color morphs were partly much lower due to our inability to identify unambiguously the color morph. The same animals used for odor extraction were also those used in the genetic analyses to test for correlations with odor. Additionally, 24 male *O*. *cornuta* were analyzed for comparison as an out-group.

### Genetic analyses

In total, we genetically analyzed 981 individuals (both males and females) of *O*. *bicornis* and *O*. *cornuta*, including males used in odor analysis. DNA was extracted using a high salt extraction protocol [33], and individuals were genotyped at six microsatellite loci developed for the species [34]. For 605 samples processed in 2009, the polymerase chain reaction (PCR) was used to amplify alleles, which were labeled radioactively using the methods described in Conrad [16].

Samples processed in 2010 (n = 376) were genotyped on an ABI 3130 DNA autosequencer. PCR amplification of polymorphic loci was undertaken in 30μl reactions containing 1μl (50–100ng) DNA, 0.1mM dNTPs, 0.42mM of fluorescently labeled forward and unlabelled reverse primer, 1mM MgCl_2_ and 0.03U Go-Taq. The PCR was run for 40 cycles at 94°C for 1min, 50°C for 1min, 72°C for 2min, with an initial 94°C denaturation step of 3 min and a final elongation step of 10 min at 72°C. Alleles were then separated on an ABI3130 DNA Analyzer, and fragment sizes were determined with GeneScan software and GeneScan 500 [Rox] length standards (Applied Biosystems, Carlsbad, California).

To ensure consistency in scoring across years, 10% of samples from 2009 were re-run and compared with the samples from 2010. Both methods generated identical genotypes.

### Statistical analyses

Relative amounts of 38 compounds found on the surface of male antennae were used in non-parametric multivariate analyses to test for population differences in odor bouquets. We performed a non-metric multi-dimensional scaling analysis (NMDS) using the program PAST [35], followed by a non-parametric multivariate statistical analysis (one-way analysis of similarities, ANOSIM) to test for significance of differences between odor bouquets (calculated as Bray-Curtis distances) of the two color morphs of males from Denmark and, in a second analysis, of differences between male odors among all nine populations. After 10000 permutations, the resulting R values were used as a measure of dissimilarity (R = 0, two groups are identical; R = 1, two groups are completely differentiated). Significance was assessed after sequential Bonferroni adjustment of p values. The main substances responsible for differentiation were obtained from a *post hoc* SIMPER analysis [36] using substances contributing more than 5% to total differentiation.

Microsatellite data were checked for null alleles, large allele dropout and scoring errors using MicroChecker version 2.2.3 [37]. GENEPOP web version 4.2 [38] was used to tests for departure from Hardy-Weinberg equilibrium (females only) and genotypic linkage disequilibrium, and to run Mantel tests of pairwise comparisons across populations. F-statistics and G_ST_ were calculated using MSA [39], which allows both male (haploid) and female (diploid) data to be incorporated into a single dataset.

As our data are hierarchical in structure (populations nested in region/country), we employed AMOVA, using Arlequin version 3.5.1.3. [40], to reveal those levels of the hierarchy at which significant genetic differentiation was found.

Partial Mantel tests were performed using the software zt [41] to test for the relationships among odor, genetic and geographic distances. Tests were performed with 10000 randomizations to check for significance of the relationship between population pairwise genetic differentiation with geographic distance, so-called ‘isolation by distance’ (F_ST_/1-F_ST_ against the natural logarithm of geographic distance [42]), and for the relationship between genetic distance and odor distance (F_ST_ against Euclidean odor distance). For the correlation between odor and population genetics, only animals included in both analyses were used unless otherwise stated.

## Results

### Chemical analyses

**Fig 1:**
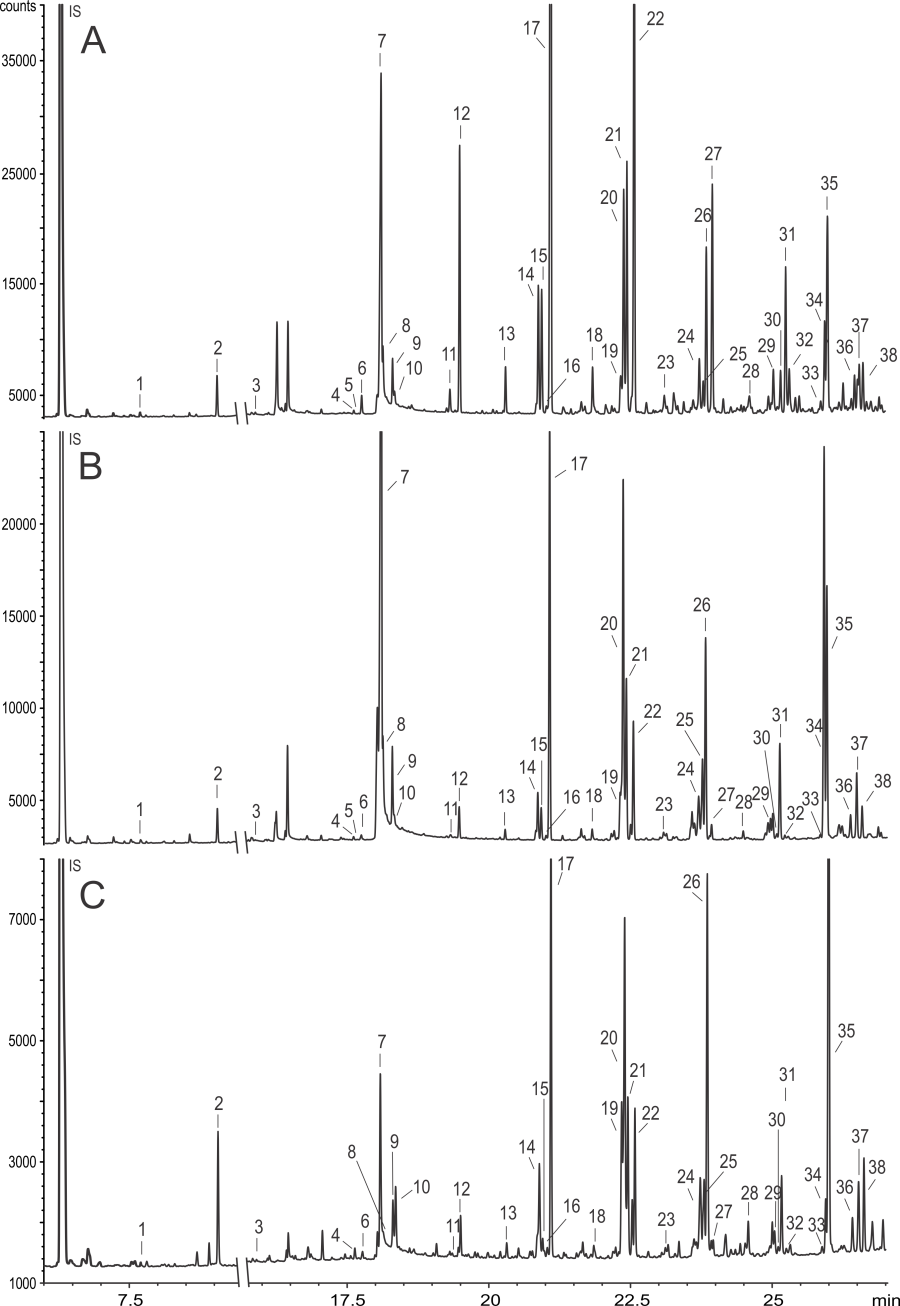
**Gas chromatogram of three male *Osmia bicornis* antennal extracts: A (England), B (Denmark) and C (Germany)**. Chemical separations were performed on a non-polar DB-5 mass spectrometry (MS) column. Peaks were quantified using GC analyses and identified using GC/MS and by comparison of gas chromatographic retention times with those of authentic reference samples. Numbered peaks correspond to the compounds listed in Table 1. Unnumbered peaks were contaminants.

We found 38 compounds on male antennae and identified 37 of them. The main compounds were alkanes and alkenes with chain lengths between 21 and 33 carbon atoms, oleic acid, its ester (ethyl oleate) and four sterols (Fig 1, Table 1). The odor bouquets differed between populations and species in relative amounts; there were no qualitative (presence/absence) differences in odors across populations and species.

**Table 1:** List of assigned compounds in the extracts of male *Osmia bicornis* antennae.

No.	compound name	No.	compound name
1	Dodecane	20	(Z)-9-Heptacosene
2	Tridecane	21	(Z)-7-Heptacosene
3	Nonadecane	22	Heptacosane
4	(Z)-9-Heneicosene	23	Octacosane
5	(Z)-7-Heneicosene	24	(Z)-11-Nonacosene
6	Heneicosane	25	(Z)-9-Nonacosene
7	Oleic acid	26	(Z)-7-Nonacosene
8	unknown (fatty acid)	27	Nonacosane
9	Ethyl linoate	28	Triacontane
10	Ethyl oleate	29	(Z)-11-Hentriacontene
11	(Z)-9-Tricosene	30	(Z)-9-Hentriacontene
12	Tricosane	31	(Z)-7-Hentriacontene
13	Tetracosane	32	Hentriacontane
14	(Z)-9-Pentacosene	33	Dotriacontane
15	(Z)-7-Pentacosene	34	24-Methylene cholesterol
16	(Z)-5-Pentacosene	35	Campesterol
17	Pentacosane	36	Tritriacontane
18	Hexacosane	37	Clerosterol
19	(Z)-11-Heptacosene	38	Δ5,24(25)-Stigmastadienol

Numbers correspond to the peaks in Fig 1. We identified 37 of 38 registered compounds by comparison of mass spectra of natural products with spectra reported in the literature and by comparison of gas chromatographic retention times with those of authentic reference samples.

First we analyzed the differences between the two sympatric *Osmia* species, where we expected differences to be greater than intraspecific differences within *O*. *bicornis*. The results of the NMDS and the one-way ANOSIM revealed a marked and significant difference in the odor bouquets of *O*. *cornuta* and *O*. *bicornis* males (R = 0.7285, P<0.05). The main substances responsible for the separation were 24-methylene cholesterol, (Z)-9-heptacosene, (Z)-11-nonacosene and (Z)-7-nonacosene (SIMPER contribution > 5%).

**Fig 2:**
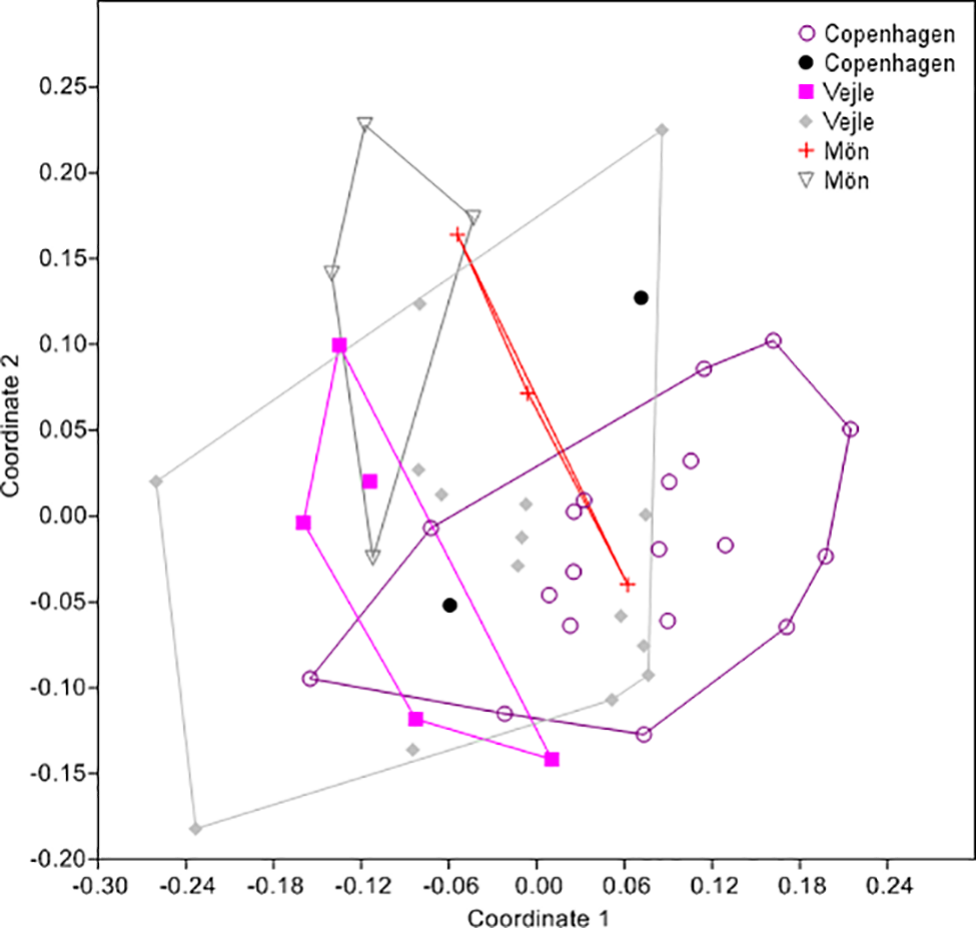
Multivariate (NMMDS) distribution of the odor bouquets of the antennae of *Osmia bicornis* males. Samples are from the three populations in Denmark (as NMMDS coordinates 1 and 2), separated according to body color. For sample sizes, see S1 Table (Bray-Curtis similarity measure; stress = 0.079; ANOSIM: R = 0.1814, P<0.05 only for pairwise comparisons: Copenhagen red with Vejle red, Vejle black with Mön black, and Copenhagen red with Mön black).

To establish if there was a difference in the odor profile between *O*. *bicornis* of different color morphs at the same location, we separated each Danish population by color morph (black or red). The results of the NMDS and the one-way ANOSIM showed that there was no significant difference between the odor bouquets of the two color morphs within a sampling locality (R = 0.1814, P>0.05) (Fig 2, S1 Table). The two color morphs did not differ in odor profile. When comparing across the three Danish localities, Mön-black males differed in odor bouquet from Vejle-black and Copenhagen-red males, and Vejle-red differed from Copenhagen-red (S1 Table); these differences do not support the idea that red and black morphs differ significantly in odor bouquet. In Denmark, differences in odor are likely due to population differentiation and are not related to color morph. For all subsequent odor analyses, we, therefore, combined data from red and black morphs at the same sampling locality.

We then analyzed differences in male odor between the nine populations of *O*. *bicornis* (excluding *O*. *cornuta*). The NMDS analysis with subsequent one-way ANOSIM revealed significant differences in the odor bouquets of the males among all populations (R = 0.3656; P<0.05 after sequential Bonferroni correction; S2 Table). The main substances responsible for the separation among populations were oleic acid, 24-methylene cholesterol, campesterol and pentacosane (SIMPER contribution > 5%; S3 Table).

Furthermore we compared differences in odor between male *O*. *bicornis* from the three regions (countries), including *O*. *cornuta* as an out-group. The NMDS analysis with subsequent one-way ANOSIM revealed significant differences in the odor bouquets of the males of regions (R = 0.3409; P<0.05 after sequential Bonferroni correction) (S2 Fig). Pairwise comparisons between populations revealed that the three populations of bees from Germany differed most from the three populations from Denmark (R = 0.2628), those from Germany differed less from populations in England (R = 0.1268), whilst populations from England and Denmark differed the least (R = 0.07217). *O*. *cornuta* differed significantly from *O*. *bicornis* from all countries, with R-values between 0.75 and 0.89.

### Genetic analysis

In total, DNA from 779 *O*. *bicornis* and 202 *O*. *cornuta* bees was successfully analyzed at 4–6 loci (S4 Table). The six microsatellites exhibited, on average, 12 alleles per locus, with the most polyallelic locus, Oru10, having 15 alleles. We found no consistent deviations from HWE or errors in scoring in our genetic data (S5 Table).

**Fig 3:**
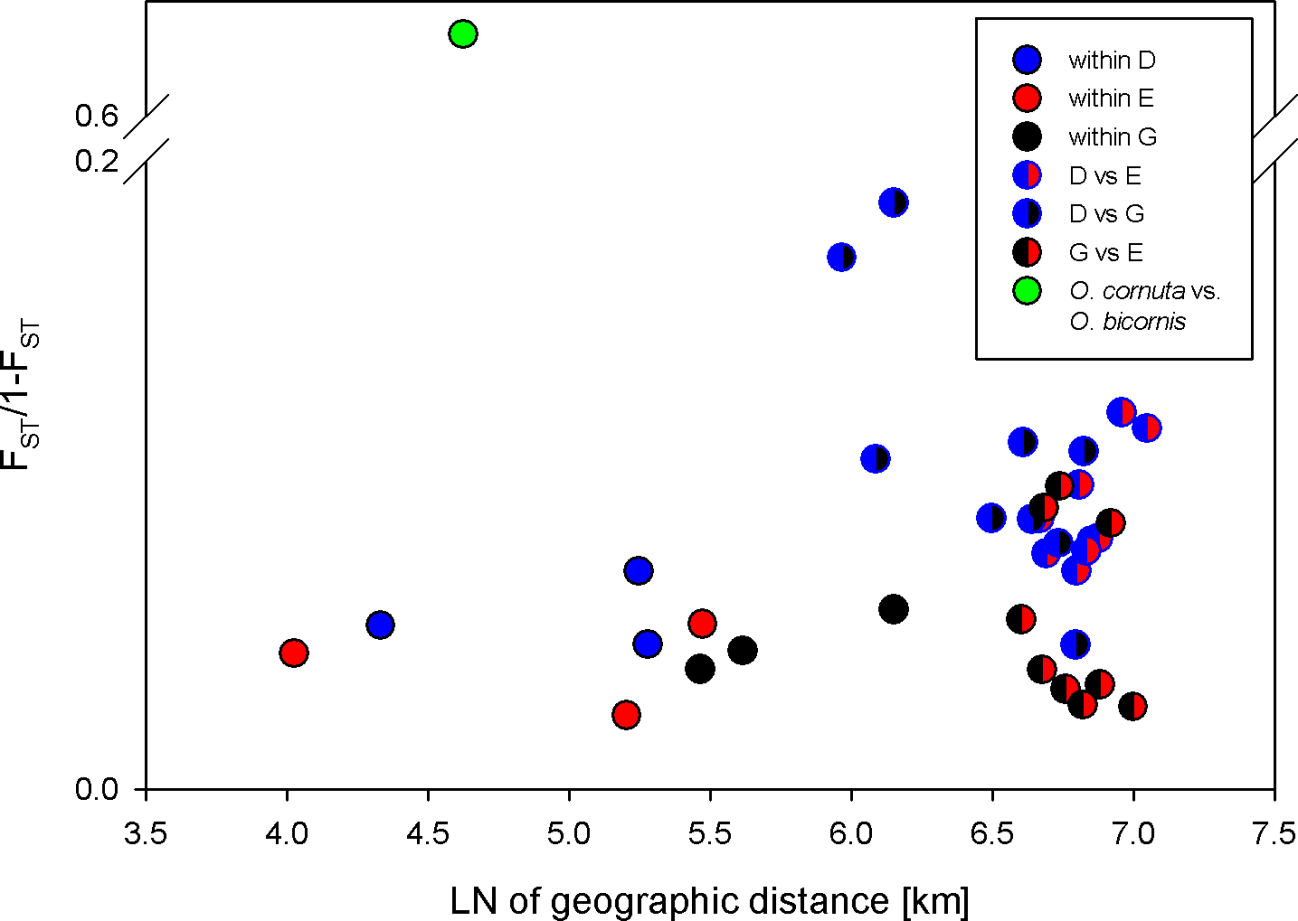
Isolation by distance relationship at 6 microsatellite loci for all 9 *Osmia bicornis* populations (a = 0.005, b = 0.011; Mantel test, P = 0.123) using Rousset’s (1997) genetic distance of F_ST_/1-F_ST_ upon (ln) distance. Danish populations in blue, English populations in red, German populations in black, and bicolored circles represent between-country comparisons. Additionally, F_ST_/1-F_ST_ is marked for *Osmia bicornis* (one population only, Constance) vs. *Osmia cornuta* (Ulm) in green as a between-species, out-group comparison.

*Osmia cornuta* differed significantly and markedly from all *O*. *bicornis* populations (P<0.005), with F_ST_ between 0.39 and 0.50 for different pairs of populations. This is well beyond intraspecific differentiation among *O*. *bicornis* populations (Fig 3).

To investigate if color morph correlated with genetic differentiation in *O*. *bicornis*, data for each Danish population were again divided into red and black color morphs. Genetic differentiation, measured as F_ST_, was insignificant for the two morphs at Mön and Copenhagen, though not at Vejle (though F_ST_ was extremely low). Differentiation among localities was significant for some morph/locality pairwise comparisons (Vejle black vs Mön red; Copenhagen red vs Mön black, Vejle red, and Vejle black; Copenhagen black vs Vejle red and black) (Table 2), suggesting we had sufficient sample sizes to detect even subtle genetic differentiation. Additionally we ran a partial Mantel test using the 6 groups (two morphs x three Danish sampling localities) with the matrices: geographic distance between sites, genetic distance between sites, and color morph. There was a significant correlation (Mantel r = 0.61) between genetic and geographical distance when controlling for color (P = 0.016), but no significant correlation between genetic distance and color when controlling for geographic distance (P = 0.147). Color morphs at a locality were weakly or not genetically differentiated; rather, genetic differentiation increased with distance. We, therefore, combined genetic data from the two color morphs within a Danish site in subsequent analyses.

**Table 2:** Pair-wise genetic differentiation (F_ST_ (below diagonal) and P (above diagonal)) of male *Osmia bicornis* bees from 3 populations in Denmark separated according to color morph.

	Mön red (n = 2)	Mön black (n = 9)	Vejle red (n = 18)	Vejle black (n = 36)	Copenhagen red (n = 43)	Copenhagen black (n = 33)
Mön red (n = 2)		0.419	0.103	**0.009**	0.547	0.243
Mön black (n = 9)	0.008		0.199	0.070	**0.032**	0.111
Vejle red (n = 18)	0.116	0.020		**0.014**	0.0018*	**0.013**
Vejle black (n = 36)	0.190	0.034	0.037		**0.000**	0.0001*
Copenhagen red (n = 43)	-0.027	0.050	0.056	0.104		0.252
Copenhagen black (n = 33)	0.046	0.027	0.042	0.102	0.005	

Significant differences are highlighted in bold for black/red differences and with an asterisk * for same color differences.

Genetic differentiation among the nine populations of *O*. *bicornis* was marked. All nine populations of *O*. *bicornis* were found to differ significantly from one another (global F_ST_ = 0.067, P<0.001), with pairwise values ranging from 0.022 between Hereford and Kent, which are geographically very close, to 0.157 between Halle and Copenhagen (S4 Table). When comparing pairwise population F_ST_ within and between countries, the mean F_ST_ within England was 0.038, within Germany it was 0.044, and within Denmark it was 0.053. Between regions, Germany and Denmark differed most, with the highest mean F_ST_ of 0.097; Germany and the England differed the least, with a mean F_ST_ of 0.050; genetically, Germany and Denmark are furthest apart, with England lying in between the two.

AMOVA, incorporating the hierarchical population structure of our sampling (for each Danish population, color morphs at a site were combined), led to a similar result. There was a significant difference at both the level of regions/countries and the level of populations (all P<0.05; S6 Table). Using pairwise comparison, we found that Germany and Denmark were the furthest apart (Fct = 0.007) and England and Germany were the least genetically differentiated (Fct<0.001). When we re-ran AMOVA including color morph as an additional level of the hierarchy, the differentiation was non-significant for color morph within a population, though populations differed significantly (S7 Table).

Isolation by distance revealed a positive trend (r = 0.011), but the correlation between F_ST_/1-F_ST_ and ln geographic distance across the nine populations of *O*. *bicornis* was not statistically significant (Mantel P = 0.123; Fig 3).

**Fig 4:**
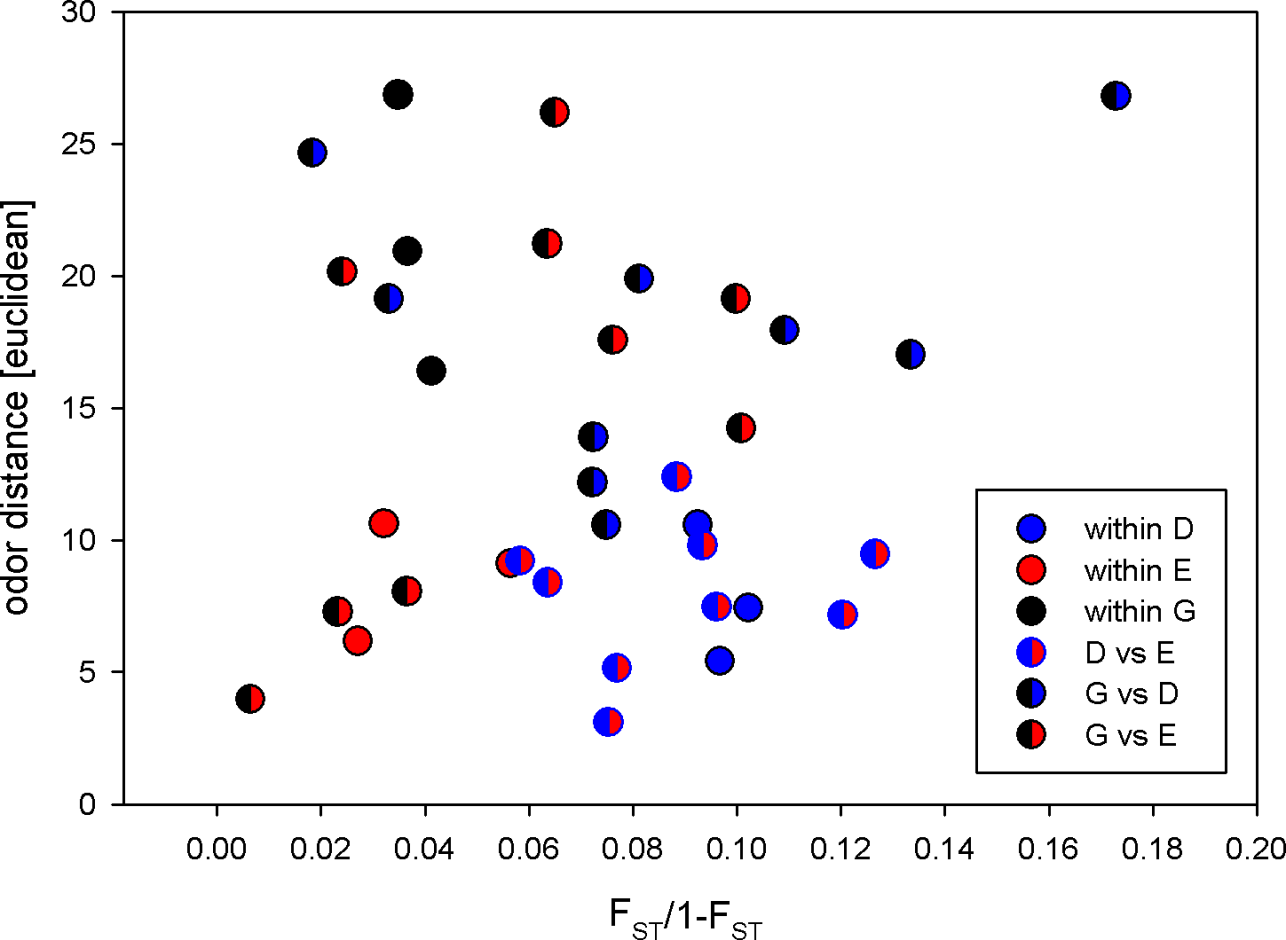
Relationship between genetic distance [F_ST_/1-F_ST_] and odor distance [as Euclidean distance] (a = 0.0694399, b = 0.00016762; Mantel test, P = 0.564) for 9 populations of *Osmia bicornis*. Danish populations in blue, English populations in red, German populations in black and bicolored circles represent between-country comparisons.

When using a Mantel test to examine the relationship between odor (Euclidean distance) and genetic differentiation (as F_ST_/1-F_ST_), we did not detect any pattern (a = 0.0645784, b = 0.00009269; Mantel P = 0.560); data points were widely scattered, suggesting differences between populations that were independent of their population genetic dissimilarity (Fig 4). An additional analysis in which we replaced F_ST_ with the population mean F_ST_ from all individuals genotyped (rather than only the genotypes of individuals used in odor analysis) was also non-significant (P = 0.210, S3 Fig).

We also ran a partial Mantel test using as variables: odor, geographic and genetic distances, to test if there was an underlying correlation between distance (as either genetic or geographic distance) and male odor profile while controlling for the other distance measure. There was neither a significant correlation between geographic and odor distances (S4 Fig), when controlling for genetic differentiation (Mantel r = -0.071, P = 0.305), nor a significant correlation between genetic distance and odor when controlling for geographic distance (Mantel r = 0.02, P = 0.474).

## Discussion

We found marked male odor differences between populations of *O*. *bicornis*, and also marked genetic differentiation, neither of which was directly associated with Peter’s’ (1978) color-based subspecies, yet there was a lack of concordance between genetic and odor signal divergence.

The results of our chemical and genetic analyses comparing males of the two subspecies of *O*. *bicornis* in Denmark show that there is neither a marked difference in odor bouquets nor genetic differentiation related to color morph. Although our sample sizes for the different color morphs were quite low at some localities, we believe the overall non-significant results to be robust because we nevertheless detected between-population differences based on the same sample sizes (in Danish color/population analyses). Based on our results, we question the classification of West European *O*. *bicornis* into two subspecies solely on color of the tip of the abdomen (a single morphological trait) because Peter’s’ (1978) intraspecific classification does not reflect other forms of biological diversification. Classification into subspecies may be prone to observed idiosyncrasies, especially if it is based on morphology alone, and it is preferable to also consider molecular differences [25,22].

The results of our chemical analyses revealed that, in addition to marked differences in the odor bouquets between the two species: *O*. *cornuta* and *O*. *bicornis*, there are also considerable intraspecific differences in the odors of males of *O*. *bicornis* from different populations, as shown by the significant differences in our ANOSIM. Information on the source population of males is, therefore, encoded in their odor bouquet. Such information that may be used by females during mate choice [43,20]. However, the compounds responsible for the differences between populations are different to the ones found to be important for female choice in our previous study [16]. In that 2010 study, metasoma extracts were used, as opposed to antennal extracts in the current study. In reality, we have little idea of which chemicals among a male’s blend are actually used by females in mate choice.

Males were more similar in odor between countries than within a country from some sites; for example, males from Kent and Vejle were more similar than were those from Mön and Vejle. These are certainly good targets for future study in order to fully understand the divergence of odor profiles. Previous studies on mate selection in *O*. *bicornis* have already shown that females preferentially mate with males from their own locality [16,17]. Mate choice based on locality has also been shown for *Colletes cunicularius*, another solitary bee, but in that case, and based on odor, males were found to prefer females from another population [44].

Selective mate choice could be an adaptation against the production of unfit hybrids that, for example, lack certain adaptations to the specific habitat or microclimate that differs between populations or regions [43,45]. This would be in accordance with Bateson’s ‘optimal outbreeding’ theory, which states that both inbreeding and outbreeding have costs and there should be a point between the two at which the sum of both is minimized [43].

Genetic differentiation between populations of *O*. *bicornis* in Europe was significant. Yet analysis of isolation by distance (IBD) revealed only a weakly positive, non-significant trend. Lack of significance might be due to low sample size (too few populations sampled) or the fact that barriers to gene flow, including the frequency of major water barriers, are not correlated with linear distance; in other bee species, major water barriers are known to limit gene flow [46,47]. We also cannot exclude the commercial movement of *O*. *bicornis* across Europe as a cause for the lack of a significant IBD signal.

It has been repeatedly shown that odor plays a predominant role in kin recognition in bees [10,11]. Kinship correlates strongly with similarities in odor, which may be important in selecting a mate [10,7], particularly in the context of kin discrimination during mating [43,48,20]. However, we found no correlation in population pairwise distances between odor similarity and genetic differentiation in *O*. *bicornis*. A possible explanation for this result is that we used the complete, complex blend of various substances found in male odors in our analyses. Individual odor components usually play different roles in an insect's behavior [49,7,1]. It is therefore possible that only some of the compounds we detected in male antennae are responsible for conveying information on genotype, while others function solely in a structural context, for example as an evaporative barrier. Odor substances with other functions could be under selection via female choice, but could also be diverging through drift.

However, it is also possible that, unlike in other bees, odor does not encode relatedness in *O*. *bicornis*, but instead these bees rely solely on the vibrational signal for information on relatedness [17]. We believe this to be unlikely, though, as these vibrational signals are not very complex and kin recognition seems to be very precise—at least for females [16,17].

Further investigation of male odor bouquets is needed to establish those substances that are relevant in female choice and that might play a role in mate recognition. The marked variation we detected between some, but not all, neighboring populations in odor bouquet may help to identify functionally relevant odor components. Using electroantennography coupled with gas chromatography (GC-EAD) in combination with bioassays with synthetic mixtures of electrophysiologically active compounds might be a useful approach to identify compounds used in intraspecific communication [50].

In conclusion, population differentiation of *O*. *bicornis* is much more complex than previously thought, and may comprise a mix of genetic drift as well as (odor) selection for local males. The discriminative mate choice by females against males from non-natal localities [16,17] and the differences in signals we report here point to a process of differentiation due to female choice, which could eventually lead to complete separation into two or more species, if genetic isolation through geography is maintained for sufficient time [22,26].

## Supporting information

S1 Fig Distribution of two subspecies of *Osmia bicornis* (red = *O*. *bicornis rufa*, green = *O*. *bicornis cornigera*) in Europe, as suggested by Peters (1987). Populations included in this study were from England (Hereford, Tonbridge and Kent: codes 1, 2 and 3 respectively), Germany (Constance, Regensburg and Halle: codes 4, 5 and 6 respectively) and Denmark (Vejle, Copenhagen and Møn: codes 7, 8 and 9 respectively).
(TIF)

S2 Fig Comparison of the odor bouquets of the antennae of males of *Osmia bicornis* from three regions of Europe, separated by NMMDS (axes are coordinate 1 and coordinate 2 of the NMMDS). For color identification, see legend (Bray-Curtis similarity measure; stress = 0.3324; ANOSIM: R = 0.3409, P<0.05 for all pair-wise comparisons).
(TIF)

S3 Fig Relationship between genetic distance and odor distance [Euclidean distance] (a = -1.1504687, b = 0.48595578; Mantel test, P = 0.20970) for males from 9 populations of *Osmia bicornis*, colored according to sampling localities. In key: D: Denmark; E, England; G: Germany.
(TIF)

S4 Fig Relationship between geographic distance and odor distance [Euclidean distance] (a = 14.4673959, b = -0.00144357; Mantel test, P = 0.809) for males from 9 populations of *Osmia bicornis*, colored according to sampling localities. In key: D: Denmark; E, England; G: Germany.
(TIF)

S1 Table Pair-wise odor differentiation (R (below diagonal) and P (above diagonal; significance after sequential Bonferroni correction) of male Osmia bicornis bees from 3 populations in Denmark separated according to color morph. Significant differences are highlighted in bold for black/red differences and with an asterisk for same color differences.
(PDF)

S2 Table NMMDS pair-wise odor differentiation, R (above diagonal), and probability of difference from zero, P, after sequential Bonferroni correction (below diagonal) of antennal extracts of male Osmia bicornis bees from 9 populations; D: Denmark; E, England; G: Germany. Significant differences are given in bold.
(PDF)

S3 Table SIMPER analyses for differences in male odor bouquets of *O*. *bicornis* populations. (XLSX)

S4 Table Pairwise F_ST_ of *Osmia bicornis* based on six microsatellite loci (above diagonal) and geographic distance (in km) (below the diagonal). All F_ST_ values were significant after sequential Bonferroni correction.
(PDF)

S5 Table Summary of allelic data for Osmia bicornis (red and black morphs) from the nine different populations. (PDF)

S6 Table Results from AMOVA analyses of O. bicornis microsatellite data using ARLEQUIN to partition the total molecular variance among different hierarchical groups. All nine populations are included and groups are defined by population membership to Germany (G), England (E) or Denmark (D).
(PDF)

S7 Table Results from AMOVA analyses of O. bicornis microsatellite data from the two different color morphs in Denmark using ARLEQUIN to partition the total molecular variance among different hierarchical groups. (PDF)
