# Supplementary figures and images for: Divergence in male sexual odor signal and genetics across populations of the red mason bee, Osmia bicornis, in Europe
Source: PLoS One. 2018 Feb 22;13(2):e0193153. doi: 10.1371/journal.pone.0193153 (PMC5823451; doi:10.1371/journal.pone.0193153)

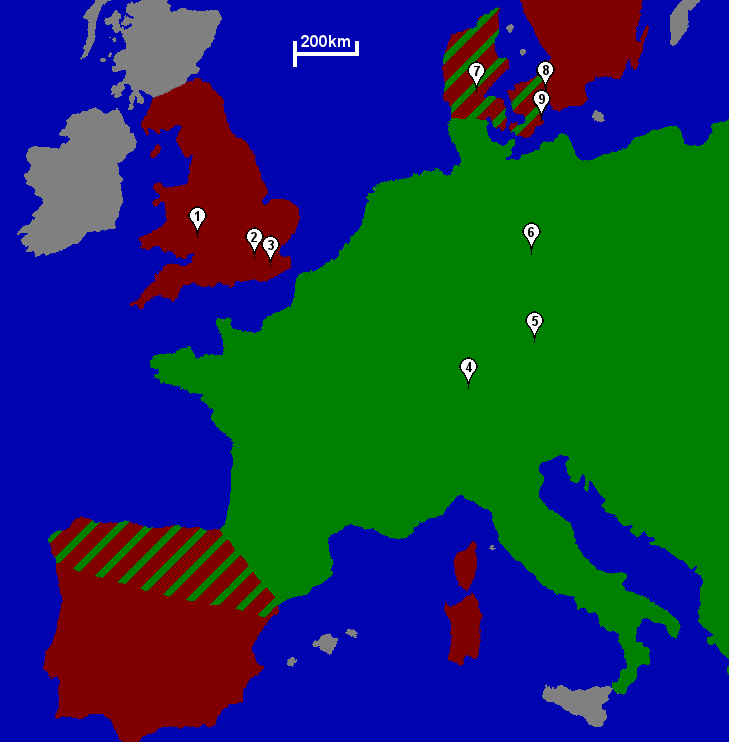

Supplement: S1 Fig — Populations included in this study were from England (Hereford, Tonbridge and Kent: codes 1, 2 and 3 respectively), Germany (Constance, Regensburg and Halle: codes 4, 5 and 6 respectively) and Denmark (Vejle, Copenhagen and Møn: codes 7, 8 and 9 respectively). (TIF) [file pone.0193153.s001.tif]
