# Supplementary material for: Divergence in male sexual odor signal and genetics across populations of the red mason bee, Osmia bicornis, in Europe
Source: PLoS One. 2018 Feb 22;13(2):e0193153. doi: 10.1371/journal.pone.0193153 (PMC5823451; doi:10.1371/journal.pone.0193153)
Supplement: S1 Table — Significant differences are highlighted in bold for black/red differences and with an asterisk for same color differences. (PDF) [file pone.0193153.s005.pdf]

Table S1 Pair-wise odor differentiation (R (below diagonal) and P (above diagonal; significance after sequential Bonferroni correction) of male Osmia bicornis bees from 3 populations in Denmark separated according to color morph. Significant differences are highlighted in bold for black/red differences and with an asterisk for same color differences.

	● Mön red (n=3)	● Mön black (n=4)	● Vejle red (n=5)	● Vejle black (n=15)	● Copenhagen red (n=19)	● Copenhagen black (n=2)
● Mön red (n=3)		0.4544	0.9067	0.3257	0.0731	0.8978
● Mön black (n=4)	-0.0556		0.1591	0.0148*	**0.0007**	0.2615
● Vejle red (n=5)	-0.2103	0.1875		0.4080	0.0265*	0.7632
● Vejle black (n=15)	0.0634	0.3670	0.0103		0.1354	0.3823
● Copenhagen red (n=19)	0.3005	0.6740	0.2522	0.0447		0.1392
● Copenhagen black (n=2)	-0.4167	0.0714	-0.1636	0.0327	0.2390	
