# Supplementary material for: Divergence in male sexual odor signal and genetics across populations of the red mason bee, Osmia bicornis, in Europe
Source: PLoS One. 2018 Feb 22;13(2):e0193153. doi: 10.1371/journal.pone.0193153 (PMC5823451; doi:10.1371/journal.pone.0193153)
Supplement: S2 Table — Significant differences are given in bold. (PDF) [file pone.0193153.s006.pdf]

Table S2 NMMDS pair-wise odor differentiation, R (above diagonal), and probability of difference from zero, P, after sequential Bonferroni correction (below diagonal) of antennal extracts of male *Osmia bicornis* bees from 9 populations; D: Denmark; E, England; G: Germany. Significant differences are given in bold.

Population	Tonbridge (E)	Hereford (E)	Kent (E)	Copenhagen (D)	Møn (D)	Vejle (D)	Regensburg (G)	Constance (G)	Halle (G)
Tonbridge (E)		**0.0001**	**0.0001**	**0.0001**	**0.0025**	**0.0006**	**0.0007**	**0.0001**	**0.0001**
Hereford (E)	0.3284		**0.0001**	**0.0001**	**0.0001**	**0.0001**	**0.0001**	**0.0001**	**0.0001**
Kent (E)	0.1993	0.2375		**0.0001**	**0.0011**	**0.0024**	**0.0001**	**0.0001**	**0.0001**
Copenhagen (D)	0.2702	0.4001	0.2462		**0.0001**	**0.0001**	**0.0001**	**0.0001**	**0.0001**
Møn (D)	0.1836	0.4205	0.2128	0.4550		**0.0031**	**0.0007**	**0.0001**	**0.0001**
Vejle (D)	0.1730	0.3354	0.1323	0.2255	0.1573		**0.0001**	**0.0001**	**0.0001**
Regensburg (G)	0.1448	0.3976	0.1716	0.5196	0.2086	0.2692		**0.0001**	**0.0001**
Constance (G)	0.4031	0.3533	0.3628	0.7052	0.3809	0.4188	0.2457		**0.0001**
Halle (G)	0.5380	0.5290	0.5441	0.7750	0.5603	0.6571	0.5720	0.6570	
