# Supplementary material for: Divergence in male sexual odor signal and genetics across populations of the red mason bee, Osmia bicornis, in Europe
Source: PLoS One. 2018 Feb 22;13(2):e0193153. doi: 10.1371/journal.pone.0193153 (PMC5823451; doi:10.1371/journal.pone.0193153)
Supplement: S4 Table — All FST values were significant after sequential Bonferroni correction. (PDF) [file pone.0193153.s008.pdf]

**Table S4 Pairwise FST of Osmia bicornis based on six microsatellite loci above diagonal and geographic distance (in km) below the diagonal. All FST values were significant after sequential Bonferroni correction.**

| | Copenhagen (D) ♂n=63; ♀n =22 | Mön (D) ♂n=17; ♀n =3 | Vejle (D) ♂n=38; ♀n =41 | Tonbridge (E) ♂n=57; ♀n =28 | Kent (E) ♂n=81; ♀n =18 | Hereford (E) ♂n=22; ♀n =22 | Constance (G) ♂n=95; ♀n =46 | Halle (G) ♂n=58; ♀n =67 | Regensburg (G) ♂n=71; ♀n =30 |
|---|---|---|---|---|---|---|---|---|---|
| Copenhagen (D) | | 0.049 | 0.064 | 0.073 | 0.073 | 0.103 | 0.097 | 0.157 | 0.099 |
| Mön (D) | 76 | | 0.044 | 0.064 | 0.070 | 0.107 | 0.072 | 0.144 | 0.079 |
| Vejle (D) | 190 | 196 | | 0.079 | 0.069 | 0.088 | 0.043 | 0.095 | 0.079 |
| Tonbridge (E) | 945 | 897 | 786 | | 0.041 | 0.049 | 0.051 | 0.082 | 0.030 |
| Kent (E) | 970 | 929 | 805 | 56 | | 0.022 | 0.036 | 0.087 | 0.026 |
| Hereford (E) | 1152 | 1052 | 906 | 238 | 182 | | 0.032 | 0.078 | 0.025 |
| Constance (G) | 920 | 842 | 893 | 738 | 794 | 975 | | 0.054 | 0.036 |
| Halle (G) | 469 | 390 | 440 | 799 | 845 | 1012 | 469 | | 0.042 |
| Regensburg (G) | 742 | 664 | 765 | 862 | 916 | 1096 | 236 | 275 | |
