# Supplementary material for: Divergence in male sexual odor signal and genetics across populations of the red mason bee, Osmia bicornis, in Europe
Source: PLoS One. 2018 Feb 22;13(2):e0193153. doi: 10.1371/journal.pone.0193153 (PMC5823451; doi:10.1371/journal.pone.0193153)
Supplement: S5 Table — (PDF) [file pone.0193153.s009.pdf]

Table S5 Summary of allelic data for *Osmia bicornis* (red and black morphs) from the nine different populations.

**Copenhagen**						
Locus	Oru10	OruS4	OruS8	OruC4	OruE5	OruA8
n ♀	17	20	22	22	19	15
n ♂	45	54	63	62	48	41
n alleles	6	7	6	5	7	3
allelic richness	5.484	6.184	5.297	4.348	6.538	2.404
Heexp	0.631	0.804	0.689	0.430	0.741	0.120
Microchecker abnormalities (females only)	null alleles	none	none	none	null alleles	none

**Tonbridge**						
Locus	Oru10	OruS4	OruS8	OruC4	OruE5	OruA8
n ♀	22	26	26	26	25	22
n ♂	37	47	49	53	50	38
n alleles	7	7	10	7	9	5
allelic richness	4.889	5.331	5.058	6.035	6.257	4.213
Heexp	0.638	0.625	0.639	0.644	0.597	0.512
Microchecker abnormalities (females only)	null alleles	null alleles	none	none	none	none

**Constance**						
Locus	Oru10	OruS4	OruS8	OruC4	OruE5	OruA8
n ♀	43	41	42	44	46	44
n ♂	87	92	91	12	95	95
n alleles	10	6	8	7	7	6
allelic richness	6.180	4.211	5.656	5.916	4.841	3.946
Heexp	0.711	0.392	0.689	0.776	0.634	0.463
Microchecker abnormalities (females only)	none	none	none	null alleles, stuttering	none	none

**Mön**						
Locus	Oru10	OruS4	OruS8	OruC4	OruE5	OruA8
n ♀	3	3	3	3	3	3
n ♂	16	17	17	17	16	15
n alleles	4	4	6	3	4	2
allelic richness	4	4	6	3	4	2
Heexp	0.657	0.529	0.731	0.318	0.556	0.292
Microchecker abnormalities (females only)	none	none	none	none	none	none

**Kent**						
Locus	Oru10	OruS4	OruS8	OruC4	OruE5	OruA8
n ♀	18	18	17	17	18	18
n ♂	74	81	80	81	81	81
n alleles	5	6	7	6	5	5
allelic richness	3.988	3.690	6.014	4.700	4.227	3.735
Heexp	0.594	0.574	0.709	0.698	0.486	0.550
Microchecker abnormalities (females only)	none	none	null alleles	none	none	none

**Halle**						
Locus	Oru10	OruS4	OruS8	OruC4	OruE5	OruA8
n ♀	64	66	66	67	59	67
n ♂	58	57	58	58	58	58
n alleles	6	5	7	4	5	4
allelic richness	4.961	4.089	5.691	3.089	3.461	3.959
Heexp	0.658	0.386	0.726	0.416	0.562	0.510
Microchecker abnormalities (females only)	none	null alleles	none	none	none	none

**Vejle**						
Locus	Oru10	OruS4	OruS8	OruC4	OruE5	OruA8
n ♀	37	41	38	40	40	39
n ♂	38	38	38	33	37	38
n alleles	7	7	9	4	7	2
allelic richness	5.122	5.288	5.89	3,804	5.687	1.952
Heexp	0.725	0.572	0.68	0.683	0.537	0.134
Microchecker abnormalities (females only)	none	none	none	none	none	none

**Hereford**						
Locus	Oru10	OruS4	OruS8	OruC4	OruE5	OruA8
n ♀	22	22	22	22	22	22
n ♂	22	22	22	22	22	22
n alleles	4	3	5	5	6	4
allelic richness	3.901	2.455	4.944	4.454	5.325	3.407
Heexp	0.650	0.518	0.701	0.745	0.749	0.505
Microchecker abnormalities (females only)	none	none	null alleles, stuttering	null alleles	none	null alleles, stuttering

**Regensburg**						
Locus	Oru10	OruS4	OruS8	OruC4	OruE5	OruA8
n ♀	30	30	29	30	30	30
n ♂	71	71	69	71	71	71
n alleles	9	4	7	4	6	5
allelic richness	5.252	3.707	5.483	3.183	4.743	4.156
Heexp	0.674	0.541	0.760	0.581	0.639	0.636
Microchecker abnormalities (females only)	none	null alleles, stuttering	null alleles, stuttering	null alleles, stuttering	none	null alleles, stuttering
