# Supplementary material for: Divergence in male sexual odor signal and genetics across populations of the red mason bee, Osmia bicornis, in Europe
Source: PLoS One. 2018 Feb 22;13(2):e0193153. doi: 10.1371/journal.pone.0193153 (PMC5823451; doi:10.1371/journal.pone.0193153)
Supplement: S6 Table — All nine populations are included and groups are defined by population membership to Germany (G), England (E) or Denmark (D). (PDF) [file pone.0193153.s010.pdf]

Table S6 Results from AMOVA analyses of O. bicornis microsatellite data using ARLEQUIN to partition the total molecular variance among different hierarchical groups. All nine populations are included and groups are defined by population membership to Germany (G), England (E) or Denmark (D).

Source of variation	% of variation	P-value
**All populations**		
Within populations	98.32	<0.05
Within countries, among populations	1.32	<0.001
Among countries	0.36	<0.001
