# Supplementary material for: Divergence in male sexual odor signal and genetics across populations of the red mason bee, Osmia bicornis, in Europe
Source: PLoS One. 2018 Feb 22;13(2):e0193153. doi: 10.1371/journal.pone.0193153 (PMC5823451; doi:10.1371/journal.pone.0193153)
Supplement: S7 Table — (PDF) [file pone.0193153.s011.pdf]

Table S7 Results from AMOVA analyses of O. bicornis microsatellite data from the two different color morphs in Denmark using ARLEQUIN to partition the total molecular variance among different hierarchical groups.

Source of variation	% of variation	P-value
**All populations**		
Within same color morph	89.77	<0.001
Within population, among color morphs	0.89	0.24
Among populations	9.34	0.13
